# A study on the association of apolipoprotein E with oxidative stress markers, neurological function, and cognitive impairment following traumatic brain injury

**DOI:** 10.3389/fneur.2025.1692712

**Published:** 2025-11-28

**Authors:** Haoyang Wang, Kun Chen, Lijun Hou

**Affiliations:** 1 Department of Neurosurgery, Shanghai Changzheng Hospital, The Second Affiliated Hospital of Naval Medical University, Shanghai, China; 2 Department of Neurosurgery, 925th Hospital of the Chinese People’s Liberation Army Joint Logistic Support Force, Guiyang, China; 3 Department of Neurosurgery, The Marine Corps Hospital of PLA, Chaozhou, China

**Keywords:** traumatic brain injury, apolipoprotein E, glutathione, malondialdehyde, cognitive function

## Abstract

**Background:**

Ttraumatic brain injury (TBI) induces oxidative stress, which contributes to neuronal damage and cognitive impairment. Apolipoprotein E (ApoE) plays a key role in neural repair and may modulate oxidative stress responses. However, the relationship between ApoE expression at different stages after TBI and oxidative stress markers, as well as its association with cognitive outcomes, remains unclear.

**Methods:**

A total of 126 patients with TBI were prospectively enrolled and stratified according to the Glasgow Coma Scale (GCS) score on admission into mild (*n* = 60), moderate (*n* = 41), and severe groups (*n* = 25). Peripheral blood samples were collected at 12 h, 24 h, and 3 days after admission to measure serum levels of ApoE, glutathione (GSH), and malondialdehyde (MDA). Cognitive function was assessed prior to discharge using the Loewenstein Occupational Therapy Cognitive Assessment (LOTCA).

**Results:**

Serum ApoE levels peaked at 24 h and slightly decreased thereafter, with overall levels increasing in proportion to TBI severity (*p* < 0.001). GSH levels progressively decreased, whereas MDA levels increased, with significant differences among the three groups (*p* < 0.001). Pre-discharge LOTCA scores were highest in the mild group and lowest in the severe group (*p* < 0.001). Spearman correlation analysis revealed that ApoE levels were negatively correlated with GSH (*r* = −0.6712) and positively correlated with MDA (*r* = 0.6934) and LOTCA scores (*r* = −0.7382) (all *p* < 0.0001).

**Conclusion:**

ApoE exhibits an injury-severity-dependent increase during the early stage of TBI, and its levels are closely associated with oxidative stress imbalance and cognitive impairment. These findings suggest that ApoE may play a critical role in both the pathological progression and neural repair following TBI.

## 1 Introduction

Traumatic brain injury (TBI) is one of the leading causes of disability and death worldwide, characterized by high incidence, high disability rate, and heavy medical burden (1). TBI not only results in extensive structural damage to brain tissue but also triggers a complex cascade of biochemical reactions, ultimately leading to long-term sequelae such as cognitive impairment, behavioral abnormalities, and reduced quality of life (2, 3). Among these, cognitive dysfunction is one of the most common and debilitating consequences of TBI. Its incidence increases significantly with injury severity, making it a critical factor in prognosis assessment and rehabilitation planning (4).

Although numerous studies have investigated the mechanisms underlying neurological dysfunction following TBI, these pathological processes remain incompletely understood. In particular, during the early stages of injury, reliable biomarkers capable of dynamically reflecting the severity of neural damage and cognitive alterations are lacking (5). Current studies have explored several promising serum biomarkers, such as ubiquitin C-terminal hydrolase L1 (UCHL1), total tau, and neurofilament light chain (NfL), to assess axonal and neuronal injury after TBI (6, 7). These markers have shown value in indicating acute neural damage; however, they mainly capture structural or cytoskeletal disruption rather than the ongoing secondary biochemical processes that drive neuronal dysfunction and repair. Among these secondary processes, oxidative stress has been recognized as a major contributor to post-traumatic neuronal damage. Glutathione (GSH) and malondialdehyde (MDA) serve as representative indicators of antioxidant capacity and lipid peroxidation, respectively, and are widely used to assess imbalances in oxidative metabolism within brain tissue (8). However, a definitive biomarker that bridges oxidative stress and neurocognitive dysfunction has yet to be established.

Apolipoprotein E (ApoE) is a key protein involved in lipid transport, neuronal regeneration, and inflammation regulation, and in recent years, it has been found to be closely associated with neural repair in Alzheimer’s disease, cerebral hemorrhage, and traumatic brain injury (9). Unlike purely structural biomarkers such as UCHL1, tau, and NfL, ApoE may reflect integrative neurobiological responses that link oxidative stress, lipid metabolism, and neuroinflammation, thereby offering a broader perspective on post-injury neural repair. Previous studies have suggested that ApoE may facilitate neural recovery after brain tissue injury by modulating neuroinflammation and oxidative stress responses. Nonetheless, its dynamic variation patterns in relation to TBI severity remain unclear, and its correlation with cognitive impairment has not been systematically validated in clinical populations (10, 11).

Given these knowledge gaps, exploring the expression patterns of ApoE in patients with TBI of varying severity, as well as its associations with oxidative stress and cognitive dysfunction, may provide potential biomarkers for individualized assessment and early intervention in TBI. In this prospective study, patients with TBI were stratified according to different Glasgow Coma Scale (GCS) scores. Dynamic monitoring of ApoE, GSH, and MDA levels was performed alongside pre-discharge cognitive assessment using the Loewenstein Occupational Therapy Cognitive Assessment (LOTCA). This approach enabled systematic analysis of the patterns and correlations of ApoE changes. This study aims to investigate the early dynamic expression of ApoE after TBI and its association with oxidative stress and cognitive impairment, thereby providing a clinical basis and research foundation for its potential role in TBI repair mechanisms.

## 2 Materials and methods

### 2.1 Baseline characteristics

Based on previous studies (12), the standardized mean difference (SMD) of TBI-related biomarkers across different severities is generally within the medium effect size range (SMD ≈ 0.5–0.8), corresponding to an effect size of approximately *f* ≈ 0.25–0.35 in a one-way analysis of variance (ANOVA) with three groups. Accordingly, an effect size of *f* = 0.30 was predetermined, with *α* = 0.05, 1 − *β* = 0.80, and *k* = 3. Sample size estimation indicated a required total of approximately *N* ≈ 111–115. Considering an anticipated dropout/exclusion rate of about 10%, the final target sample size was set at *N* = 126.

A total of 126 hospitalized patients with TBI admitted to our hospital between December 2021 and December 2023 were enrolled. Patients were stratified based on their first available GCS score after admission (13) into the following groups: mild group (*n* = 60, GCS 13–15), moderate group (*n* = 41, GCS 9–12), and severe group (*n* = 25, GCS 3–8). Grouping and baseline characteristics are presented in Table 1. The study protocol was approved by the Institutional Ethics Committee of Shanghai Changzheng Hospital, The Second Affiliated Hospital of Naval Medical University (No. SHCZ20250420), and written informed consent was obtained from all participants or their legal guardians.

**Table 1:** Comparison of baseline characteristics among patients with different severities of traumatic brain injury [ $\bar{x}$ ± s, *n* (%), M (P_25_, P_75_)].

Factors		Mild group (*n* = 60)	Moderate group (*n* = 41)	Severe group (*n* = 25)	*F*/*H*/ $\chi^{2}$	*p*
Age		50.23 ± 5.20	50.58 ± 4.36	49.92 ± 5.28	0.66	0.52
Sex	Male	36 (60.0%)	23 (56.0%)	17 (68.0%)	0.92	0.63
	Female	24 (40.0%)	18 (43.9%)	8 (32.0%)		
Degree of education	Junior high school or below	17 (28.33%)	10 (23.81%)	6 (24.0%)	0.56	0.76
	Senior high school or above	43 (71.67%)	32 (78.05%)	19 (76.0%)		
GCS score		6 (6, 7)	10 (9, 11)	13 (13, 14)	110.45	<0.001

Data are presented as mean ± SD, *n* (%), or median (P_25_, P_75_). Differences among groups were analyzed using one-way ANOVA for normally distributed data, Kruskal–Wallis test for non-normally distributed data, and $x^{2}$ test for categorical variables.

Inclusion criteria: (1) met the diagnostic criteria for TBI (14) and confirmed by imaging; (2) presented within 24 h after injury; (3) age >18 years; (4) complete clinical data.

Exclusion criteria: (1) history of diagnosed cognitive impairment or central nervous system disease (e.g., dementia, active epilepsy, prior cranial surgery); (2) severe uncontrolled medical comorbidities (e.g., decompensated cardiac, hepatic, or renal failure); (3) active malignancy or acute infection.

### 2.2 Methods

Peripheral venous blood samples were collected at 12 h (T1), 24 h (T2), and day 3 (T3) after admission, preferably in a fasting state; if fasting was not feasible, the collection status was recorded. Samples were centrifuged at 3,000 rpm for 10 min within 30 min of collection to separate serum, which was aliquoted and stored at −80 °C until analysis.

Serum ApoE (ab108813, Abcam, Shanghai, China), GSH (BB-4711, Bestbio, Shanghai, China), and MDA (6074ES50, Yeasen, Shanghai, China) levels were quantified using enzyme-linked immunosorbent assay (ELISA) kits according to the manufacturers’ protocols. For each assay, 100 μL of serum was used for quantitative measurement.

Before discharge (target 7–14 days; assessment performed only when patients were awake and able to cooperate), cognitive function was assessed once using the LOTCA (7). Examiners were uniformly trained and blinded to the laboratory results.

### 2.3 Statistical analysis

Statistical analysis was performed using IBM SPSS Statistics software, version 21.0 (IBM Corp., Armonk, NY, United States). Measurement data were first tested for normality (Shapiro–Wilk test) and homogeneity of variance (Levene’s test). Normally distributed and homoscedastic data were expressed as mean ± standard deviation $\left(\bar{x}\pm s\right)$ and compared among groups using one-way ANOVA with Bonferroni *post hoc* correction. Normally distributed but heteroscedastic data were analyzed using Welch’s ANOVA with Games-Howell *post hoc* correction. Non-normally distributed data were expressed as median and interquartile range [M (P_25_, P_75_)] and compared among groups using the Kruskal–Wallis test with Dunn–Bonferroni *post hoc* correction. Categorical data were expressed as number and percentage [*n* (%)] and compared among groups using the $x^{2}$ test. For repeated measures, repeated measurement ANOVA was applied under normality assumptions, with Greenhouse–Geisser correction applied when sphericity was violated. For non-normally distributed repeated measures, the Friedman test was used, with Bonferroni correction applied for pairwise comparisons. Correlation analyses were performed using Spearman’s rank correlation test. All tests were two-sided, and a *p*-value <0.05 was considered statistically significant.

## 3 Results

### 3.1 Serum ApoE levels at different time points after admission

**Figure 1:**
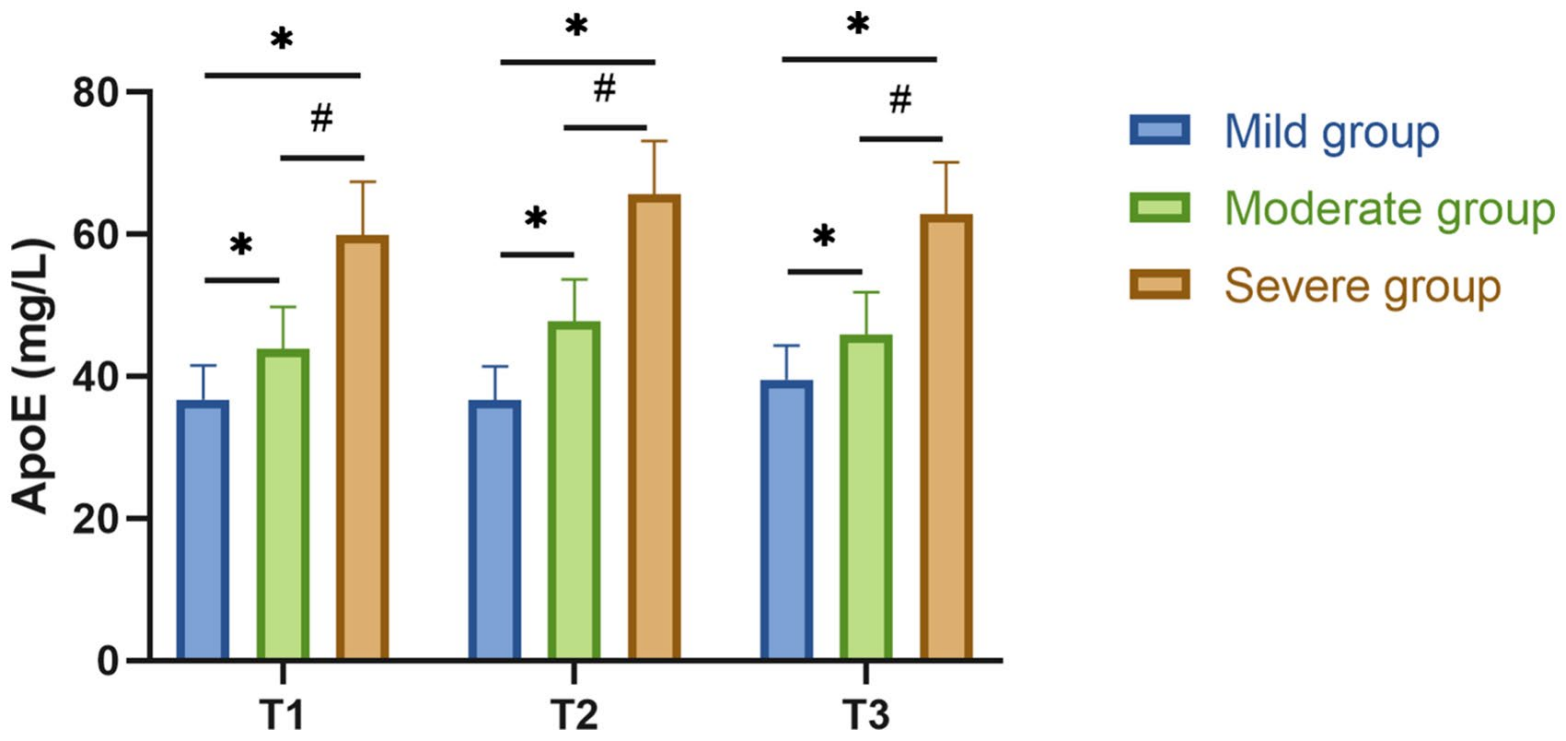
Comparison of serum ApoE levels among TBI groups at different time points. Serum ApoE concentrations (mean ± SD) were measured at 12 h (T1), 24 h (T2), and 3 days (T3) after admission in patients with mild, moderate, and severe traumatic brain injury. Statistical analysis was performed using one-way ANOVA followed by pairwise *post hoc* tests. ^*^p < 0.05 vs. mild group; ^#^*p* < 0.05 vs. moderate group.

Serum ApoE levels increased proportionally with TBI severity, with statistically significant differences among the three groups at each time point (*F* = 139.88, 89.89, and 145.18 for T1–T3, respectively; all *p* < 0.001). Cross-sectional comparisons at the same time point showed that both the moderate and severe groups had significantly higher ApoE levels than the mild group, and the severe group had higher levels than the moderate group (all *p* < 0.05). Within-group longitudinal comparisons indicated that ApoE levels in all groups peaked at 24 h (T2), showing a significant increase compared with 12 h (T1) (*p* < 0.05). By day 3 (T3), ApoE levels had slightly declined compared with T2 but remained significantly higher than T1 (*p* < 0.05). This pattern was consistent across all groups, and the overall magnitude of increase was positively associated with injury severity (Table 2 and Figure 1).

**Table 2:** Serum ApoE levels at different time points after admission in patients with varying TBI severity [ $\bar{x}$ ± s, M (P_25_, P_75_)].

Group	ApoE (mg/L)		
	T1	T2	T3
Mild group (*n* = 60)	36.68 ± 4.85	36.06 (33.22, 40.59)	39.48 ± 4.85^^-^
Moderate group (*n* = 41)	43.85 ± 5.94^*^	48.12 (42.82, 51.39)^*^^	45.84 ± 5.98^*-^
Severe group (*n* = 25)	59.84 ± 7.54^*#^	67.29 (62.66, 69.67)^*#^^	62.84 ± 7.26^*#^-^
*F*/*H*	139.88	89.89	145.18
*p*	<0.001	<0.001	<0.001

Data are presented as mean ± SD or median (P_25_, P_75_). Intergroup comparisons were performed using one-way ANOVA or Kruskal–Wallis test, followed by *post hoc* pairwise comparisons. Intragroup differences across time points were analyzed using repeated-measures ANOVA or Friedman test as appropriate. ^*^p < 0.05 versus mild group; ^#^*p* < 0.05 versus moderate group; ^^^*p* < 0.05 versus T1; ^-^*p* < 0.05 versus T2.

### 3.2 Oxidative stress markers (GSH and MDA) at different time points after admission

**Figure 2:**
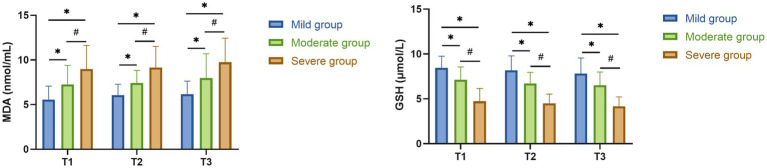
Serum oxidative stress marker levels at different time points after admission in patients with mild, moderate, and severe TBI. Serum malondialdehyde (MDA, nmol/mL) and glutathione (GSH, μmol/L) concentrations (mean ± SD) were determined at 12 h (T1), 24 h (T2), and 3 days (T3) after admission. Comparisons among TBI severity groups were performed using one-way ANOVA followed by *post hoc* pairwise tests. ^*^p < 0.05 vs. mild group; ^#^*p* < 0.05 vs. moderate group.

GSH levels decreased progressively with increasing injury severity at all time points, whereas MDA levels increased accordingly. Differences among the three groups were statistically significant (GSH: *F* = 67.14, 61.42, and 58.34 for T1–T3, respectively; all *p* < 0.001; MDA: *F* = 23.79, 28.88, and 24.06 for T1–T3, respectively; all *p* < 0.001). *Post hoc* comparisons showed that GSH levels in the moderate and severe groups were significantly lower than those in the mild group, with the severe group also significantly lower than the moderate group. Conversely, MDA levels were highest in the severe group, significantly exceeding those in the moderate and mild groups (all *p* < 0.05). Within-group comparisons revealed that GSH levels declined significantly from 24 h compared with 12 h and further decreased by day 3, whereas MDA levels rose at the same pattern. These temporal trends were consistent across all groups (all *post hoc* adjusted *p* < 0.05) (Table 3 and Figure 2).

**Table 3:** Comparison of oxidative stress marker levels (GSH and MDA) at different time points after admission among TBI groups of varying severity [ $\bar{x}$ ± s, M (P_25_, P_75_)].

Group	GSH (μmol/L)			MDA (nmol/mL)		
	T1	T2	T3	T1	T2	T3
Mild group (*n* = 60)	8.47 ± 1.28	8.17 ± 1.62^^^	7.47 (6.32, 9.05)^^-^	5.56 ± 1.51	6.04 ± 1.24^^^	6.15 ± 1.48^^-^
Moderate group (*n* = 41)	7.12 ± 1.43^*^	6.72 ± 1.24^*^^	6.58 (5.35, 7.61)^*^-^	7.25 ± 2.14^*^	7.42 ± 1.41^*^^	7.98 ± 2.71^*^-^
Severe group (*n* = 25)	4.73 ± 1.43^*#^	4.50 ± 1.03^*#^^	4.49 (3.52, 4.76)^*#^-^	8.98 ± 2.64^*#^	9.15 ± 2.37^*#^^	9.74 ± 2.71^*#^-^
*F*/*H* value	67.14	61.42	58.34	23.79	28.88	24.06
*p*-value	<0.001	<0.001	<0.001	<0.001	<0.001	<0.001

Data are presented as mean ± SD or median (P_25_, P_75_). Intergroup differences were analyzed using one-way ANOVA or Kruskal–Wallis test, followed by *post hoc* pairwise comparisons. Intragroup differences across time points were assessed using repeated-measures ANOVA or Friedman test, as appropriate. T1 = 12 h after admission; T2 = 24 h after admission; T3 = 3 days after admission. ^*^p < 0.05 versus mild group; ^#^*p* < 0.05 versus moderate group; ^^^*p* < 0.05 versus T1; ^-^*p* < 0.05 versus T2.

### 3.3 Cognitive function before discharge (LOTCA)

LOTCA scores before discharge differed significant in cognitive function levels among the three groups (*F* = 353.18, *p* < 0.001). The mild group had the highest scores, followed by the moderate group, with the severe group scoring lowest. Pairwise comparisons further demonstrated that both the moderate and severe groups scored significantly lower than the mild group (all *p* < 0.05), and the severe group scored significantly lower than the moderate group (*p* < 0.05) (Table 4).

**Table 4:** Comparison of LOTCA scores before discharge among patients with different severities of TBI [ $\bar{x}$ ± s].

Group	LOTCA scores
Mild group (*n* = 60)	72.62 ± 5.12
Moderate group (*n* = 41)	58.61 ± 3.79^*^
Severe group (*n* = 25)	45.20 ± 3.87^*#^
*F* value	353.18
*p*-value	<0.001

Data are presented as mean ± SD. Differences among groups were analyzed using one-way ANOVA followed by *post hoc* pairwise comparisons. ^*^p < 0.05 versus mild group; ^#^*p* < 0.05 versus moderate group.

### 3.4 Correlation analysis

**Figure 3:**
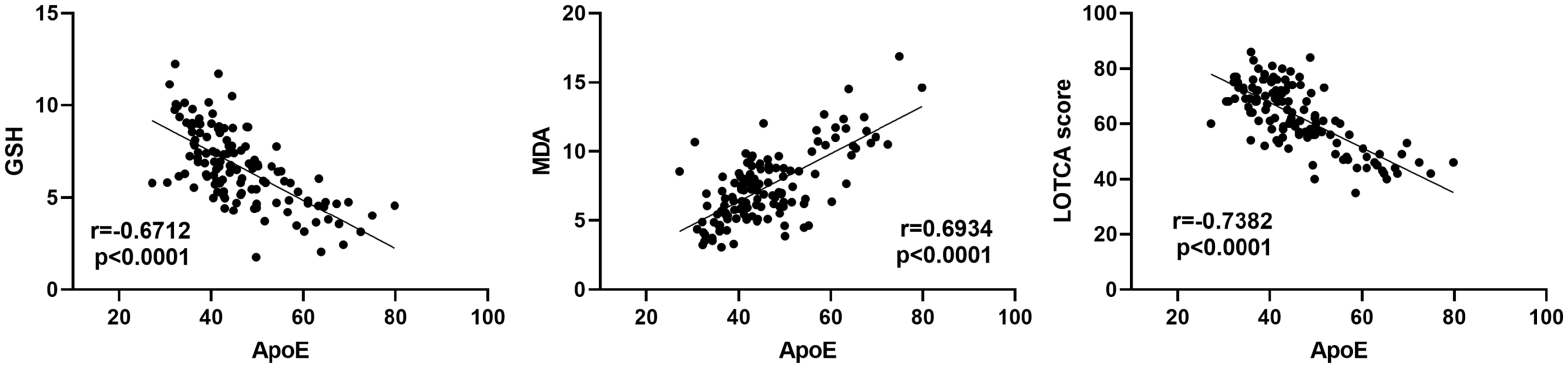
Correlation analysis between serum ApoE levels and oxidative stress markers (GSH, MDA) and cognitive function (LOTCA scores) in patients with TBI.

On day 3, serum ApoE levels were significantly negatively correlated with GSH levels (*r* = −0.6712, *p* < 0.0001), positively correlated with MDA levels (*r* = 0.6934, *p* < 0.0001), and negatively correlated with LOTCA scores before discharge (*r* = −0.7382, *p* < 0.0001) (Table 5 and Figure 3).

**Table 5:** Correlation analysis between serum ApoE levels and oxidative stress markers (GSH, MDA) and cognitive function (LOTCA scores) in patients with TBI.

Factors	ApoE	
	*r*	*p*
GSH	−0.6712	<0.0001
MDA	0.6934	<0.0001
LOTCA scores	−0.7382	<0.0001

Correlation coefficients were calculated using Pearson’s rank correlation test.

## 4 Discussion

This study systematically analyzed the dynamic changes of ApoE during the acute phase of TBI and its relationship with oxidative stress and cognitive function, aiming to explore the potential role of ApoE in the pathological progression and neural repair processes of TBI from both clinical and mechanistic perspectives. We found that serum ApoE levels increased in an injury-severity-dependent manner, peaking within 24 h after admission, followed by a slight decline, yet remaining significantly above baseline on day 3. This “early elevation-short-term maintenance-gradual decline” pattern was consistent across all injury severities, indicating that ApoE elevation reflects an early systemic response to neuronal damage. Moreover, its significant correlations with oxidative stress indicators (GSH, MDA) and cognitive function (LOTCA score), suggesting that ApoE may not only be involved in the metabolic response after TBI, but also be closely associated with oxidative imbalance and cognitive impairment (15).

ApoE, primarily synthesized by astrocytes, is a key lipid transport protein in the central nervous system. It redistributes cholesterol and phospholipids and plays essential roles in maintaining cell membrane stability, axonal regeneration, and synaptic plasticity (16, 17). Previous studies have shown that ApoE exerts neuroprotective effects after neural injury by regulating lipid metabolism, attenuating neuroinflammation, and reducing oxidative damage (18–22). These mechanisms help maintain neuronal homeostasis under stress conditions and are closely aligned with the present findings. The rapid increase in serum ApoE within 24 h after admission, followed by partial decline but sustained elevation at day 3, likely reflects an acute compensatory reaction to membrane disruption and inflammatory activation after brain injury (9, 16, 23, 24). This observation supports the interpretation that ApoE upregulation represents an adaptive, early-phase response aimed at restoring cellular integrity and lipid balance in the injured brain.

Of note, ApoE levels were moderately negatively correlated with GSH and positively correlated with MDA, indicating that its elevation may not reflect enhanced repair capacity, but rather a pathological response signal (25, 26). GSH and MDA represent antioxidant capacity and lipid peroxidation status, respectively, and their temporal trends were highly coupled with ApoE changes, showing a “worsening with disease severity” pattern after TBI (5, 27). This close coupling suggests that ApoE elevation may serve as a biological indicator of oxidative imbalance, reflecting the organism’s attempt to counteract redox stress while simultaneously mirroring the extent of metabolic disturbance. However, under sustained oxidative stress, ApoE may undergo conformational or functional modifications that reduce its receptor affinity or even confer pro-inflammatory effects (28–30), thereby diminishing its reparative role. Such bidirectional behavior echoes the “double-edged sword” property of ApoE, whose functional impact likely depends on the surrounding redox environment. Further analysis revealed that ApoE levels were significantly negatively correlated with LOTCA scores, indicating that higher ApoE levels were closely associated with worse cognitive function, reinforcing its potential as a clinical biomarker for functional impairment in TBI (15, 31). This finding underscores the clinical relevance of ApoE dynamics: while its early rise may represent a compensatory metabolic response, sustained or excessive elevation could signal persistent oxidative burden and impaired neural recovery. Notably, LOTCA provides a multidimensional evaluation of cognitive function with strong biological validity (32), thereby supporting the robustness of this correlation.

Compared with previous studies, the main contribution of this work lies in clarifying, through dynamic observation and stratified analysis, the correlation among ApoE, oxidative stress, and cognitive function in patients with different severities of TBI. This provides clinical-level evidence for ApoE as an acute-phase biomarker of TBI. In contrast to most prior research that has centered on ApoE genotypes (such as the ε4 allele) or long-term outcomes, systematic evidence regarding acute phase protein changes has been limited (5, 31). Our results fill this gap, suggesting that increased ApoE more likely reflects injury burden and metabolic response intensity, rather than directly indicating the degree of “neural repair” (9, 33). These findings suggest that ApoE serves as a molecular bridge linking early oxidative-inflammatory processes with clinical recovery, and that its biological role may shift from adaptive to detrimental depending on the redox microenvironment (34, 35).

Clinically, ApoE holds promise as a biomarker for early risk stratification and prognosis in TBI. Combined with GSH and MDA, it could form an “oxidative-metabolic” assessment axis, guiding identification of high-risk patients and individualized interventions. Especially in severe TBI patients, the strong negative correlation between ApoE and cognitive impairment indicates that early elevation of ApoE may predict poor neurological outcomes, serving as a reference for early rehabilitation and clinical management. Moreover, ApoE testing is well-established, with convenient sample collection, and can be routinely performed on standard biochemical platforms in most hospitals, supporting good feasibility and translational potential. If combined with ApoE genotype information in the future, a gene–protein–phenotype three-dimensional predictive model could be constructed, potentially enhancing the role of ApoE in precision medicine management of TBI.

Nevertheless, several limitations of this study must be acknowledged. First, this was a single-center prospective observational study with a limited sample size, particularly in the severe group (*n* = 25), which may lead to insufficient statistical power and restrict generalizability. Second, we only measured peripheral ApoE levels, without incorporating dynamic changes from cerebrospinal fluid, ApoE isoforms, or oxidative modifications, which limits mechanistic interpretation. Additionally, we did not perform a comparative analysis with other established TBI biomarkers such as glial fibrillary acidic protein (GFAP), ubiquitin carboxy-terminal hydrolase L1 (UCH-L1), neurofilament light chain (NfL), or Tau, which have been widely recognized for their diagnostic and prognostic value. The absence of such benchmarking limits our ability to determine whether ApoE provides incremental predictive information beyond currently validated biomarkers. Future studies integrating ApoE into multimarker panels alongside these proteins may better define its unique contribution to TBI characterization and outcome prediction. Importantly, peripheral ApoE is predominantly synthesized by the liver and does not readily cross the blood–brain barrier, whereas CNS ApoE is largely produced by glial cells, undergoes distinct post-translational modifications, and plays key roles in downregulating neuroinflammation, promoting synaptic plasticity, and modulating excitotoxicity after injury. Thus, changes in peripheral ApoE cannot be directly extrapolated to CNS ApoE dynamics, and our findings should be interpreted cautiously in this context. Nevertheless, it remains possible that peripheral ApoE may indirectly influence CNS outcomes through modulation of systemic inflammatory mediators or by gaining access to the brain after disruption of the blood–brain barrier in severe TBI. Third, long-term follow-up was not included, and LOTCA assessments were limited to a single pre-discharge evaluation, preventing assessment of whether ApoE changes predict long-term cognitive recovery. Future studies should adopt longitudinal neuropsychological evaluations, imaging analyses, and molecular subtyping to better clarify the temporal sequence of ApoE changes and their prognostic implications. In addition, potential confounders such as comorbidities, nutritional status, and medication use were not controlled for, which should be addressed in future research. Finally, we did not include a non-CNS trauma control group (e.g., orthopedic injury without brain involvement), which limits our ability to determine whether ApoE elevation is specific to TBI rather than reflecting a non-specific acute-phase response. Future studies incorporating such comparator groups are warranted to strengthen the interpretation of ApoE specificity. Moreover, the absence of ApoE genotyping represents another biologically relevant limitation. ApoE is a polymorphic protein encoded by three major alleles (ε2, ε3, and ε4), and accumulating evidence indicates isoform-dependent effects on oxidative stress, lipid metabolism, and neural repair after CNS injury. Individuals carrying the ε4 allele, in particular, exhibit impaired lipid transport, reduced antioxidant capacity, and attenuated neuroregenerative responses compared with ε3 carriers. Beyond these classical isoforms, regulatory noncoding variants (such as rs405509 and rs192879175) have been shown to differentially modulate ApoE transcriptional activity, thereby influencing circulating and CNS ApoE concentrations. Given the critical involvement of ApoE in cholesterol homeostasis and redox regulation, the lack of genotype and lipid profile data precludes a deeper understanding of how genetic and metabolic heterogeneity may shape post-traumatic ApoE responses. Future research integrating ApoE genotyping, noncoding variant analysis, and detailed lipidomic profiling will be essential to delineate isoform-specific and transcriptionally mediated pathways through which ApoE modulates oxidative injury, neuroinflammation, and recovery trajectories following TBI.

## 5 Conclusion

This study characterized the dynamic changes of ApoE during the acute phase of TBI, highlighting its close association with oxidative stress and cognitive function. ApoE elevation not only paralleled injury severity, but may also serve as a key biomarker for assessing oxidative stress and functional impairment risk. These findings provide a foundation for mechanistic studies and clinical application of ApoE in TBI. Future research should integrate genotyping, inflammatory factor profiling, and long-term functional outcomes to expand its translational potential in individualized TBI management.

## Data Availability

The original contributions presented in the study are included in the article/supplementary material, further inquiries can be directed to the corresponding author.
